# Protein fingerprints of cultured CA3-CA1 hippocampal neurons: comparative analysis of the distribution of synaptosomal and cytosolic proteins

**DOI:** 10.1186/1471-2202-9-36

**Published:** 2008-04-10

**Authors:** Valeria Corti, Yovan Sanchez-Ruiz, Giovanni Piccoli, Andrea Bergamaschi, Carlo V Cannistraci, Linda Pattini, Sergio Cerutti, Angela Bachi, Massimo Alessio, Antonio Malgaroli

**Affiliations:** 1 Proteome Biochemistry, San Raffaele Scientific Institute, Milan, Italy; 2 Mass Spectrometry, San Raffaele Scientific Institute, Milan, Italy; 3 Neurobiology of learning units, San Raffaele Scientific Institute, Milan, Italy; 4 Università Vita-Salute San Raffaele, Milan, Italy; 5 Department of Biomedical Engineering, Polytechnic University, Milan, Italy

## Abstract

**Background:**

All studies aimed at understanding complex molecular changes occurring at synapses face the problem of how a complete view of the synaptic proteome and of its changes can be efficiently met. This is highly desirable when synaptic plasticity processes are analyzed since the structure and the biochemistry of neurons and synapses get completely reshaped. Because most molecular studies of synapses are nowadays mainly or at least in part based on protein extracts from neuronal cultures, this is not a feasible option: these simplified versions of the brain tissue on one hand provide an homogeneous pure population of neurons but on the other yield only tiny amounts of proteins, many orders of magnitude smaller than conventional brain tissue. As a way to overcome this limitation and to find a simple way to screen for protein changes at cultured synapses, we have produced and characterized two dimensional electrophoresis (2DE) maps of the synaptic proteome of CA3-CA1 hippocampal neurons in culture.

**Results:**

To obtain 2D maps, hippocampal cultures were mass produced and after synaptic maturation, proteins were extracted following subfractionation procedures and separated by 2D gel electrophoresis. Similar maps were obtained for the crude cytosol of cultured neurons and for synaptosomes purified from CA3-CA1 hippocampal tissue. To efficiently compare these different maps some clearly identifiable reference points were molecularly identified by mass spectrometry and immunolabeling methods. This information was used to run a differential analysis and establish homologies and dissimilarities in these 2D protein profiles.

**Conclusion:**

Because reproducible fingerprints of cultured synapses were clearly obtained, we believe that our mapping effort could represent a simple tool to screen for protein expression and/or protein localization changes in CA3-CA1 hippocampal neurons following plasticity.

## Background

Synapses are complex structures that regulate neuronal communication and mediate virtually all functions of the nervous system. They are highly polarised structures because of the asymmetric distribution of cytosolic and membrane proteins, such as ion channels and signaling molecules. Such a polarized organization is essential for the vectorial transport of conventional neurotransmitter molecules and other types of messenger molecules. The presynaptic compartment or presynaptic bouton contains synaptic vesicles and all other components of the fusion machinery involved in vesicular exo- endo-cytosis and its regulation (see [1] for review). Synaptic vesicles greatly increase the total amount of lipid bilayer contained in the presynaptic bouton and proportionally the number of membrane associated molecules. In the postsynaptic compartment membrane clusters of neurotransmitter receptors are highly enriched and in excitatory terminals these are embedded in a characteristic electron-dense structure called the postsynaptic density. In this area neurotrasmitter receptors are tightly associated directly or indirectly with other proteins subserving as scaffolding elements, regulators of signalling and of exo-endocytosis (see [2] for review).

It is well established that neuronal activity produces a multiplicity of "molecular "changes in pre- and post-synaptic compartments to sustain a continuum of structural and functional synaptic modifications. Among the activity-dependent synaptic changes the most well known example is represented by NMDA- dependent long-term potentiation (LTP) [3]. LTP includes both short-term changes in synaptic strength, within minutes and hours, and long-term modifications, taking place over the course of days. Beyond three hours, LTP seems critically dependent on protein and mRNA synthesis as suggested by its suppression with translational and transcriptional inhibitors (see [4] for review). These and other results suggest that potentiated synapses must differ in some key molecular component(s) from control or unpotentiated terminals. These molecular components should be either synthesized at the neuronal soma and then specifically recruited by immature or potentiated synapses (see [5] for review) or made in situ from locally stored mRNA molecules (see [6]for review). The question that immediately comes to mind is how can we follow the time-dependent protein changes occurring at potentiated synapses in an efficient way? Knowing the exact temporal profile of these changes would be important not only to reveal mechanistic relations among different groups of synaptic molecules but also to define the exact temporal order of known plasticity processes. Unfortunately at the present time no simple method is available for a global comparison of protein profiles which are characteristic of synapses in different functional states. The availability of detailed 2DE maps of synapses would facilitate this task, an approach that was pioneered by Bliss laboratory [7].

In the last few years several proteomic analyses of brain synapses, aimed at the identification of either novel synaptic components or interacting protein complexes (see [8] for review) or their post-translational modifications [9,10] have been published. Unfortunately in most of these studies, synaptic samples purified from various brain areas were used [11-14]. The variability and complexity of these 2D protein maps indicates that it would be important to obtain a more simplified and homogeneous synaptic material to be able to use this information to estimate protein expression changes at synapses. For example, subcellular extracts of synapses (synaptosomes) from brain always contain variable degrees of contamination with spurious material originating from glial cell membranes, myelin vesicles and other non neuronal sources. Also, even when a reliable reference map enriched with proteins associated with synaptic function could be obtained, it would be essential to obtain in parallel maps from the other subcellular compartments. Because many proteins do shuttle back and forward from the cell soma or from the dendrites to the post-synaptic apparatus (see [2]for review), to understand who is migrating and in which direction protein pedigrees should be available for both synaptosomes and cytosol. Importantly proteomic maps of synapses should be available for those systems where synaptic plasticity can be easily and homogeneously induced and analysed with the modern tools of molecular and cellular biology. In this context, cultured neurons, and in particular cultured CA3-CA1 hippocampal neurons from neonatal rat brain would represent a very feasible system. These neuronal cells form synapses in vitro with well-developed spines and display various forms of synaptic plasticity including NMDA receptor-dependent LTP [15,16] and synaptic scaling or synaptic adaptation [17,18]. Because many observations about synaptic plasticity were made in hippocampal cultures, and because they allow precise manipulation and monitoring of synaptic function, they are now a widely used system for studies of the molecular mechanisms of synaptic plasticity. The purification of pure synaptic fractions from dissociated CA3-CA1 hippocampal cultures, obtained from neonatal rat brain, would be facilitated because these cultures contain few glial cells and almost no other non neuronal material. At least in principle, these cultures would be the ideal target for a detailed proteomic analysis of neurons and synapses but the paucity of the protein material that can be extracted represents an obstacle. Thus, the optimization of solubilization and purification procedures for these protein mixtures would be required.

Here we present the generation of 2DE maps from cultured CA3-CA1 synapses and neuronal cytosol. Our results show that synapses can indeed be purified from cultures with a greater level of purity than when brain hippocampal tissue is used. The 2D maps that we obtained provided reproducible fingerprints of these subcellular structures, hence we foresee that our work could provide the basis for future studies in hippocampal cultures aimed at the molecular understanding of synaptic plasticity.

## Results

### Generation of 2D electrophoresis maps of synaptosomes from CA3-CA1 hippocampal cultures

We have previously employed a classical fractionation approach for the purification of synaptic membranes and cytosol from CA3-CA1 hippocampal synapses [19]. The starting material was obtained from dissociated cultures, which were prepared from neonatal rat brain (P4-P5) after microdissection of the CA3-CA1 region. In the present study the same source of synaptic material was used to obtain pure synaptosomes and generate 2DE maps of this subpopulation of hippocampal synapses. To evaluate the extent of enrichment in synaptic proteins and the degree of contamination with glia and myelin proteins, subcellular fractions were analyzed by standard electron microscopy (EM) and by western blotting (see Additional file 1). These two methods showed that the degree of sample pureness was indeed much greater in protein extracts from cultures than in extracts from CA3-CA1 hippocampal tissue ([19] and unpublished data).

**Figure 1:**
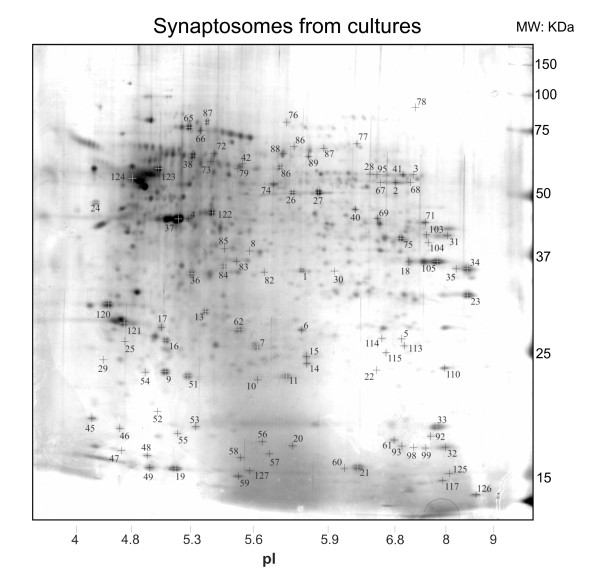
**Silver stained two-dimensional gel electrophoresis reference map of synaptosomes from CA3-CA1 neuronal cultures**. The numbered spots were chosen for further characterization by tryptic digestion and MALDI MS. Among these: 3, synapsin II; 29, synaptophysin; 30, sintaxin 1A; 45, β-synuclein; 56, α-synuclein (for complete list see table 1).

In figure 1 is presented the 2D reference map of synaptosomes purified from CA3-CA1 cultured neurons. Around 1000 spots were detected in the size range 15–150 kDa with isoelectric point (*pI*) values comprised between 4 and 9. The general appearance of these 2D maps, the molecular weight (MW) and *pI *distributions, the number and the relative intensity of individual protein spots were all reproducible in different experiments (See below; n = 12, number of gels used for this study to. This number includes n = 9 analytical and preparative gels, and n = 3 gels used for Western blot analysis; the number of spots detected in the best analytical silver stained gels which were used for differential expression analysis is n = 1154 ± 231, mean ± sem; n = 3 gels.)

### Identification of individual reference spots in synaptosomal 2D maps

To obtain a set of reference points to efficiently put in frame and compare different gels, we selected individual protein spots and subjected them to trypsin digestion and MALDI-TOF MS analysis. These spots were chosen among those that apparently gave the most characteristic features of synaptosomes maps so that the whole map area was well represented (n = 105 spots; see methods for details). The list of reference proteins here used is presented in table 1. A number of specialized pre-synaptic molecules, ion channels and transporters, adhesion and cytoskeletal elements molecules, components of various metabolic pathways, mitochondria components, chaperones, proteosomal subunits, receptors and signaling molecules could be identified among these reference points (table 1). In parallel experiments, 2D gels were blotted and immunolabelled with specific antibodies to localize additional references based on well known synaptic markers (n = 3; see method for details; table 1). Despite the finding that sometime 2D gels could present slightly distorted migration pattern, with variation in the spatial distribution of protein spots and MW – *pI *markers, the reference spots could always be recognized and allowed a straightforward comparison of gels from different cell culture preparations (n = 9). Based on this reproducibility we can then conclude that 2D maps of synaptosomes from cultures can be reliably obtained.

**Table 1:** List of proteins identified in synaptosomes and crude cytosol from hippocampal culture or tissue

Spot number	Accession number*	Protein name	S tis	S cul	C cul	Method of detection
**Cytoskeletal and their interacting Proteins(CK)**						
4	P60711	Actin, cytoplasmic	x	x	x	2DE-MS
37	P63259	Actin, cytoplasmic 2	x	x	x	2DE-MS
48	Q61274	Alpha-cardiac actin [Fragment]	x	x		2DE-MS
100, 101, 123	P68370	Tubulin alpha-1 chain	x	x	x	2DE-MS
36, 39, 47,124	P04691	Tubulin beta chain	x	x	x	2DE-MS
22	P37805	Neuronal protein 25	x	x		2DE-MS
53	P13668	Stathmin	x	x	x	2DE-MS
119	P13668	Stathmin			x	2DE-MS
55	Q63228	Glia maturation factor beta	x	x	x	2DE-MS
33	P45592	Cofilin, non-muscle isoform	x	x	x	2DE-MS
92	Q9R0P5	Destrin	x	x	x	2DE-MS
50	P63029	Translationally controlled tumor protein	x			2DE-MS
125	P62963	Profilin I		x	x	2DE-MS
127	Q9EPC6	Profilin II		x	x	2DE-MS
**Metabolism(M)**						
18, 105, 106	P04797	Glyceraldehyde-3-phosphate dehydrogenase	x	x	x	2DE-MS
71	P16617	Phosphoglycerate kinase, testis specific	x	x	x	2DE-MS
96	P10719	ATP synthase beta chain, mitochondrial	x	x	x	2DE-MS
11	P31399	ATP synthase D chain, mitochondrial	x	x		2DE-MS
26, 27	P04764	Alpha enolase	x	x	x	2DE-MS
84	P11980	Pyruvate kinase, M2 isozyme	x	x	x	2DE-MS
78	Q9ER34	Mitochondrial aconitase [Precursor]	x	x	x	2DE-MS
2, 67	P10860	Glutamate dehydrogenase, mitochondrial [Precursor]	x	x	x	2DE-MS
34, 35, 64	P04636	Malate dehydrogenase, mitochondrial [Precursor]	x	x		2DE-MS
49	P11240	Cytochrome c oxidase polypeptide VA	x	x	x	2DE-MS
8, 85	Q99NA5	NAD+specific isocitrate deihydrogenase a-subunit	x	x		2DE-MS
83	P42123	L-lactate dehydrogenase B chain	x	x	x	2DE-MS
5	P48500	Triosephosphate isomerase	x	x	x	2DE-MS
31, 103	P05065	Fructose-biphosphate Aldolase A	x	x	x	2DE-MS
40	P09117	Fructose-bisphosphate aldolase C	x	x	x	2DE-MS
41, 68	Q9D0K2	Succinil-CoA:3-ketoacid-coenzyme A transferase 1, mitochondrial	x	x	x	2DE-MS
56	Q05982	Nucleoside diphosphate kinase A	x	x	x	2DE-MS
61	P19804	Nucleoside diphosphate kinase B	x	x	x	2DE-MS
69	P09606	Glutamine synthetase	x	x	x	2DE-MS
70	P25809	Creatine kinase, ubiquitous mitochondrial [Precursor]	x		x	2DE-MS
75	P13221	Aspartate aminotransferase cytoplasmatic	x	x	x	2DE-MS
122	P07335	Creatine kinase, B chain	x	x	x	2DE-MS
1, 82	O88989	Cytosolic malate dehydrogenase	x	x	x	2DE-MS
80	P97532	3-mercaptopyruvate sulfurtransferase	x			2DE-MS
90	P04764	Alpha-enolase	x		x	2DE-MS
104	Q80YG1	Phosphoserine aminotransferase		x	x	2DE-MS
113, 114	P48500	Triosephosphate isomerase		x	x	2DE-MS
16	P19234	NADH-ubiquinone oxidoreductase 24 kDa subunit	x	x	x	2DE-MS
**Synaptic components (SY)**						
72, 76, 86, 87	P47942	Dihydropyrimidinase related protein-2 (drp-2)	x	x	x	2DE-MS
89	Q62952	Dihydropyrimidinase related protein-3 (drp-3)	x	x	x	2DE-MS
3	Q63537	Synapsin II	x	x	x	2DE-MS
29	P07825	Synaptophysin	x	x		WB
30	P32851	Syntaxin 1 A-E	x	x		WB
46	P37377	Alpha-synuclein	x	x		2DE-MS
45	Q63754	Beta-synuclein	x	x		2DE-MS
73	P25286	Vacuolar proton translocating ATPase subunit a isoform 1	x	x		2DE-MS
9, 51	P31044	Phosphatidylethanolamine-binding protein 1	x	x	x	2DE-MS
19	P55051	Fatty acid-binding protein, brain	x	x	x	2DE-MS
126	P11030	Acyl-CoA-binding protein		x	x	2DE-MS
128	P62775	Myotrophin			x	2DE-MS
**Receptors, Ion Channel and adhesion molecules (I)**						
23, 63	Q6P9W9	Voltage-dependent anion-selective channel protein 1	x	x	x	2DE-MS
66	P50516	Vacuolar ATPsynthase catalytic subA	x	x		2DE-MS
**Vesicle Transport and Recycling (T)**						
97	P46462	Transitional endoplasmic reticulum ATPase	x		x	2DE-MS
25	P63012	Ras-related protein rab-10	x	x	x	WB
28	Q62824	Exocyst complex component Sec8		x		WB
**Protein Fate (synthesis, folding, modification and destination) (PF)**						
38	P63039	60 kda heat shock protein, mitochondrial precursor (hsp60)	x	x	x	2DE-MS
65	P63017	Heat shock 70 kDa protein-ps1	x	x	x	2DE-MS
102	P07901	Heat shock protein [Fragment]			x	2DE-MS
44	P48721	Stress-70 protein, mitochondrial [Precursor]	x	x	x	2DE-MS
17	Q00981	Ubiquitin carboxyl-terminal hydrolase isozyme L1	x	x		2DE-MS
15	P20108	Mitochondrial thioredoxin-dependent peroxide reductase	x	x	x	2DE-MS
52	Q9DB15	39S ribosomal protein L12, mitochondrial	x	x		2DE-MS
6	P52555	Endoplasmic reticulum protein ERp29	x	x	x	2DE-MS
32, 98, 99	P10111	Peptidyl-prolyl cis-trans isomerase A	x	x	x	2DE-MS
42, 43, 79	P11598	Protein disulfide isomerase A3	x	x	x	2DE-MS
77	O35814	Stress induced phosphoprotein 1	x	x	x	2DE-MS
88	P11983	T complex polypeptide 1b	x	x	x	2DE-MS
21	P55053	Fatty acid-binding protein, epidermal	x	x	x	2DE-MS
59	P07483	Fatty acid-binding protein, heart	x	x	x	2DE-MS
57	Q9EQX9	Ubiquitin-conjugating enzyme e2	x	x	x	2DE-MS
108, 109	P04905	Glutathione S-transferase Yb-1			x	2DE-MS
107	P04904	Glutathione S-transferase Yc-1			x	2DE-MS
112	P08010	Glutathione S-transferase Yb-2			x	2DE-MS
111	P08009	Glutathione S-transferase Yb-3			x	2DE-MS
116	P14942	Glutathione S-transferase alpha-4			x	2DE-MS
110	Q63716	Peroxiredoxin-1		x	x	2DE-MS
54	P35704	Peroxiredoxin-2	x	x	x	2DE-MS
93	Q9R063	Peroxiredoxin-5, mitochondrial [Precursor]	x	x	x	2DE-MS
94	Q63347	26S protease regulatory subunit 7			x	2DE-MS
115	P17220	Proteasome subunit alpha type 2		x	x	2DE-MS
91	Q9JHW0	Proteasome subunit beta type 7 [Precursor]	x		x	2DE-MS
**Signalling Proteins (S)**						
24	P10354	Chromogranin A	x	x		WB
74	P50399	RAB GDP dissociation inhibitor beta-2	x	x	x	2DE-MS
10	P60766	Cell division control protein 42 homolog	x	x		2DE-MS
60	P70349	Histidine triad nucleotide-binding protein	x	x	x	2DE-MS
81	P14669	Annexin III	x			2DE-MS
117	Q62658	FK506-binding protein 1°		x	x	2DE-MS
120	P62260	14-3-3 protein epsilon		x	x	2DE-MS
121	P63102	14-3-3 protein zeta/delta		x	x	2DE-MS
62	P62994	Growth factor receptor bound protein 2	x	x		2DE-MS
**Other (O)**						
12	O35264	Platelet-activating factor acetylhydrolase IB subunit beta	x		x	2DE-MS
13	P67779	Prohibitin	x	x	x	2DE-MS
7	O35244	Antioxidant protein 2	x	x	x	2DE-MS
20	P07632	Superoxide dismutase [Cu-Zn]	x	x	x	2DE-MS
58	Q9CPT4	IL25	x	x	x	2DE-MS
14	O88767	CAP1 PROTEIN	x	x	x	2DE-MS
118	P01041	Cystatin B			x	2DE-MS
95	Q8CDL9	Similar to hypothetical protein	x	x	x	2DE-MS

S: synaptosome; C: crude cytosol; cul: culture; tis: tissue; 2DE-MS: two-dimensional electrophoresis and MALDI-TOF mass spectrometry; WB: Western blot; * Swiss-Prot database; Spot numbers correspond to the spot numbers indicated in Fig. 1 and 4.

### Comparing maps of synaptosomes from cultures with those from hippocampal tissue

**Figure 2:**
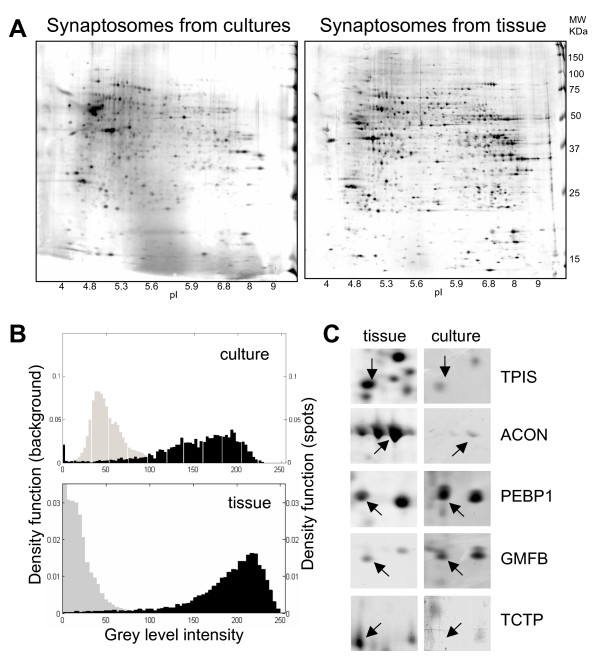
**Comparative analysis of protein profiles from synaptosomes obtained from CA3-CA1 cultured cells and CA3-CA1 hippocampal brain tissue**. A) Exemplar protein profiles of the synaptosomes from CA3-CA1 cultures (left), and CA3-CA1 hippocampal tissue (right), separated by two-dimensional gel electrophoresis and silver stained. B) Distribution of spot intensities (black histograms) and background noise (grey histograms) from the same 2D gels in panel A from cultures (upper panel) and hippocampal tissue (lower panel). C) Some exemplar proteins whose expression differed between the two samples. TPIS: Triosophosphate isomerase; ACOM: mitochondrial aconitase; PEBP1: Phosphatydilethanolamine-binding protein 1; GMFB: Glial maturation factor beta; TCTP: translationally controlled tumor protein (see also table 1).

To evaluate differences between cultured synapses and those that can be purified from mature brain tissue we obtained 2D protein profiles of CA3-CA1 hippocampal tissue synaptomes (n = 3; Figure 2). To allow a more straightforward comparison of both set of maps, the same reference spots selected for cultured synapses were searched in tissue maps and subjected to MALDI-TOF analysis. We could recognize and sequence 73 of the original reference points while 8 additional reference points were added for a total of 81 reference points for the tissue map (see list in table 1). Using common reference spots we could compare the two sets of maps. Both the macroscopic appearance and the number of spots were found to be reasonably conserved (number of spots in CA3-CA1 hippocampal tissue n = 1406 ± 378, mean ± sem; n = 3) (Figure 2, panel A). This is somehow expected based on the common origin of these samples and on the equivalent developmental age and degree of maturation of synapses (P4-P5 rats plus 14 days of development in vitro for cultured synapses; P21 rats for brain tissue). Despite this general similarity, when these 2D maps were compared in greater details, some clear differences in protein profiles and in relative protein abundances emerged, with only ~50% of spots that were found to have similar expression levels in both sets of gels. The latter finding cannot be accounted by neither a difference in total protein content (proteins loads were comparable) nor by a decreased in the signal to noise ratio in gels of cultured synapses. The distribution of spot intensities and gel background were indeed always well separated from each other and the results were found to similar in both groups (Figure 2, panel B). This excludes a simple detection problem as a source of discrepancy. In figure 2 (panel C) are presented some magnified regions of these 2D maps to illustrate some cases of proteins whose expression differed between cultures and tissue up to exemplars well below the threshold for detection in either one of the two data sets. Some of the proteins listed in table 1 are likely to be protein contaminants from non neuronal sources. Based on their volumes in 2D maps, these protein contaminants were clearly more prominent in extracts from brain tissue, an expected finding according to the abundance of non synaptic structures seen in tissue synaptosomes preparations by EM analysis (unpublished data; see discussion).

### Generation of 2D map from neuronal crude cytosol: comparative analysis with the synaptic proteome

**Figure 3:**
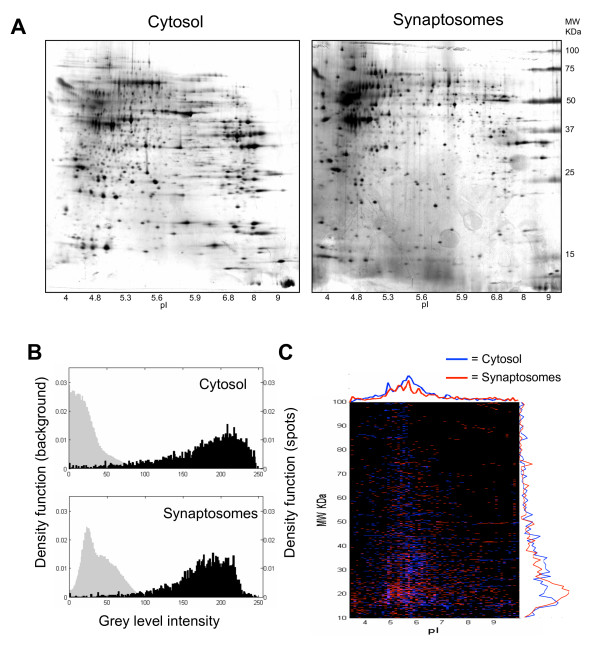
**Comparative analysis of protein profiles from synaptosomes and crude cytosolic fractions obtained from CA3-CA1 cultured cells**. A) Exemplar protein profiles of crude cytosolic (left) and synaptosomes (right) fractions, separated by two-dimensional gel electrophoresis and silver stained. B) Distributions of spot intensities (black histograms) and background noise (grey histograms) from the same 2D gels of panel A from cytosol (upper panel) and synaptosomes (lower panel). C) Digital representation of the superimposition of the two protein profiles (cytosol, blue; synaptosomes, red) after spot detection (see methods for details).

In parallel experiments we obtained two-dimensional gel electrophoresis maps of crude cytosolic extracts from CA3-CA1 hippocampal cultures. Because of the very low density of glial cells and because of the very large extent of neuronal dendrites in these cultures, most of this material is likely to derive from cytosol and endoplasmic reticulum of dendrites. Across different experiments the general appearance and the number and the distribution of protein spots from cytosolic extracts was found to be reproducible (n = 12, number of gels used including n = 9 analytical and preparative gels, and n = 3 gels used for Western blot analysis; the number of spots detected in the best analytical silver stained gels which were used for differential expression analysis is n = 1099 ± 236, mean ± sem; n = 3 gel). In figure 3 (panel A) two exemplar maps of cytosolic and synaptic extracts of CA3-CA1 hippocampal cultures are compared. Already at the visual level, clear differences in the spatial distribution of spots can be appreciated, indicating that we are dealing with two largely different protein sets. To better compare the spatial distribution of protein spots in these two maps, the detected protein spots were color coded and spatially superimposed (Figure 3, panel C). The digital superimposition shows the enrichment in low *pI *proteins for synaptosomes and in low MW proteins for cytosolic fractions. As indicated in Figure 3 (panel B), these results cannot be attributed to different signal to noise ratios because in the two sets of gels distributions of spot intensities were similar and well separated from background noise.

### Sequencing of individual reference spots from cytosolic 2D maps

**Figure 4:**
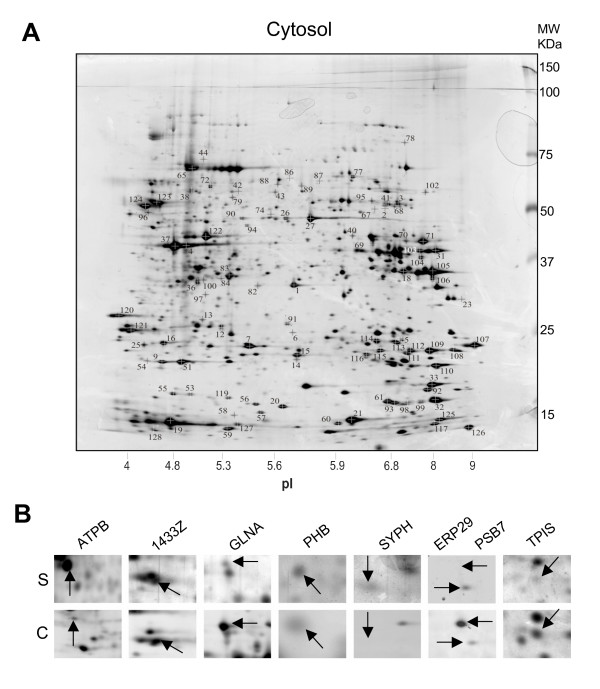
**Silver stained two-dimensional gel electrophoresis reference map of the crude cytosolic fraction from CA3-CA1 neuronal cultures and comparison with profiles from synaptosomes**. A) 2D reference map from crude cytosolic fraction stained using silver. The numbered spots were chosen for further characterization by tryptic digestion and MALDI MS. C) Some examples of proteins whose expression differed between the two samples. ATPB, ATP synthase β chain, mitochondrial;1433Z, 14-3-3 protein zeta/delta; GLNA, Glutamine synthetase; PHB, Prohibitin; SYPH, Synaptophysin; PSB7, Proteasome subunit β type 7; ERP29, Endoplasmic reticulum protein ERp29; TPIS, Triosephosphate isomerase.

When comparing synaptic and crude cytosolic maps of CA3-CA1 cultures, we searched for the same protein spots used as markers of the synaptosomal maps. We could recognize and identify by MALDI-TOF MS 89 of the original reference points while 16 novel additional reference points, more easily appreciated in cytosolic maps, were sequenced for a total of 105 reference points (Figure 4) (see list in table 1). The identified proteins belong to different functional groups including: soluble components of synapses (synapsin II), metabolic and mithocondria components, chaperones, elements of the ubiquitin-proteosomal system, cytoskeletal elements, signaling or regulatory molecules (table 1). As expected very few trans-membrane proteins were present except for some components of the endoplasmic reticulum and of the endosomal compartment. Several proteins listed in table 1 were present in both synaptic and cytosolic fractions. On the contrary, many cases of proteins, with clear differences in relative expression levels, including some which were selectively absent in either one set of maps were clearly identified (Figure 4, panel B; additional file 2 and 3). In particular proteins belonging to metabolism and protein fate (synthesis, folding, modification and destination) functional groups were specifically expressed or the expression level was increased in the crude cytosolic fraction (additional file 2). On the contrary, synaptic components were mostly detected only in the synaptosomal fraction (additional file 2). These results suggest that many of these elements might be steadily required in one or both cellular districts or might shuttle between cytosol and synapses.

## Discussion

Hippocampal synapses are very dynamic structures whose size, overall morphology and activity are reflections of previous activation history. Since Bliss and Lomo pioneering work [3] most studies on synaptic plasticity in the hippocampus have been performed either in- vivo or on the hippocampal slice preparation. More recently, dissociated cultures from either the whole hippocampus [17,20-25] or from the CA3-CA1 region [15,16,18,26,27] have become an increasingly popular system for synaptic plasticity studies. By rigorously purifying synaptic fractions from CA3-CA1 hippocampal mature cultures, we provide here reference 2D maps for their synaptic components. Although a map of whole rat hippocampal neuronal cells has been previously reported [28], at the present time, the 2D maps presented here are the only available comprehensive proteomic overviews of the synaptosomal and cytosolic fractions of cultured rat hippocampal neurons. Therefore, we believe that this information could be very useful for future comparative studies of protein expression changes at neurons and synapses in different physiological and pathological conditions where protein expression is modulated either by synthesis/degradation or translocation between different cellular compartments. In this study we set up the optimal strategy for protein solubilization to capture more of the insoluble proteins which are usually excluded from detection. The fact that only some of the well known elements of synapses were detected in the present analysis relates to the choice of limiting sequencing just to the most visible spots, i.e. the most highly abundant proteins. Our goal was to establish 2D fingerprints of synapses and cytosol to direct future identification of areas where following synaptic plasticity protein expression or migration is altered more than to provide a complete proteomic analysis of all synaptic components. Therefore the protein spots we selected could only be the most abundant components otherwise these maps would not be useful as references to screen from 2D gels of standard experiments where, because of the few culture dishes involved, the amount of proteins available is very low.

If the purpose is to evaluate the biological significance of protein changes either expression changes, post-translational modifications or subcellular delocalizations, then experiments on dissociated neuronal cultures clearly offer several advantages. The most important is the possibility to obtain neuronal protein material almost uncontaminated from glial cells, myelin and brain blood vessels components, which are common contaminants of tissue subcellular fractions. This is indicated by the degree of enrichment with synaptic and glial cell markers in culture synaptosomes [19] which was higher than in tissue synaptosomes. This was also confirmed by ultrastructural imaging of culture and tissue synaptic fraction at the EM level (unpublished data). Our 2D maps from cultures and tissue confirmed this view. Then the observed divergence between in-vitro and in-vivo 2D maps of synaptic proteins presumably reflects the expected larger degree of enrichment in neuronal material when fractions are purified from hippocampal cultures. Part of this difference might also arise from the more heterogeneous population of synapses found in the brain tissue where multiple neurochemical phenotypes are present. It cannot be excluded that some of these differences depend on the reduced expression of those synaptic proteins which are induced by the *in-situ *interactions between glial and neuronal cells, a process whose extent must be greatly diminished in culture dishes. Because the animal sacrifice is known to induce intense synaptic activation, some of the discrepancies found might at least in theory arise from fast protein changes, either post-translational modifications or protein translocation, which could take place in those few minutes that follow animal decapitation and cessation of blood flow. In this respect, it is clear that experiments run on neuronal cultures permit a more controlled acquisition of the resting proteome of synapses.

## Conclusion

The availability of 2D maps for CA3-CA1 hippocampal cultures, a very popular model system for synaptic plasticity studies, with identified landmark proteins for both the neuronal cytosol and synapse, will certainly help quantifying protein expression changes occurring in both cellular compartments in different physiological states. In the present paper we also present maps of the crude cytosol of cultured neurons, gathered with the idea of screening for proteins localized both in the cytosol and in the endoplasmic reticulum. At least in theory the availability of these fingerprints should permit revealing those changes arising from massive translocations of specific proteins between the cytosol and ER of cell somato/dendrites to the synapse. We hope that the generation of these detailed fingerprints might represent a useful reference for uncovering some aspects of activity-dependent synaptic modifications at CA3-CA1 hippocampal synapses

## Methods

### Hippocampal cell cultures

Postnatal CA3-CA1 hippocampal cultures were prepared from P4-5 neonatal rats essentially as previously described [16]. Briefly, the hippocampal regions, CA3-CA1, were removed from rat brains and neurons recovered by enzymatic digestion (trypsin type XI, 10 mg/ml, DNAse I type IV, 0.5 mg/ml) followed by mechanical dissociation. Cells were cultured in Minimal Essential Medium, glucose 0.6%, glutamine 1 mM, NaHCO_3 _2.4 g/l, bovine transferrin 100 mg/ml, insulin 25 mg/ml, fetal calf serum 5% and plated at a density of 50,000 per dish onto petri dishes (Falcon, 30 mm) coated with polyornithine and Matrigel (Collaborative Research). Cultures were maintained at 37°C in a 95% air/5% CO_2 _humidified incubator. The culture media was replaced every 3 to 4 days. From the second day in culture, the media was supplemented with cytosine-D-arabinofuranoside (5 μM). Synaptosomes were prepared from hippocampal neurons after 14 days in culture. The culture media and the fetal calf serum were obtained from GIBCO (Italy), all other chemicals were obtained through Sigma (Italy).

### Synaptosomes preparation, protein purification and solubilization

Synaptosomes were obtained from control CA3-CA1 hippocampal cultures and tissue (P21 rats), the latter after surgical microdissection of the CA3-CA1 region. For culture experiments, Petri dishes (3 cm diameter) were briefly washed with a phosphate buffered saline (4°C) and then neuronal cells were detached in 0.32 M sucrose (100 μl/Petri dish) by gentle scraping in the presence of a protease inhibitor cocktail. Neurons were homogenized using a small capacity (2 ml) Teflon/glass homogenizer in a final volume of 500 μl (5 Petri dishes for each cycle) at 4 C. After all Petri dishes were processed, the total homogenate was transferred into small centrifuge tubes (Beckman Ultraclear 344057 Centrifuge tubes) and centrifuged at 1200 g/10 × min (4°C) using a fixed angle rotor (Beckman TL100 Ultracentrifuge). The resultant supernatant was then centrifuged again at 12.500 g/20 × min (4°C) to obtain a mitochondria- and synaptosome-enriched pellet and the crude cytosolic fraction (containing also microsomes). Similar methods were used to prepare the synaptosomal and crude cytosolic fractions from CA3-CA1 hippocampal tissue utilized for the generation of 2D maps employed in differential analysis. In some experiments with brain tissue, to set up the method for protein extraction, synaptosomes were further purified using multiple differential gravity centrifugation [29]. At the end of the procedure, synaptosomal and crude cytosolic fractions were collected and quickly frozen in small aliquots. The amounts of proteins were quantified by Bradford assay (Bio-Rad, Hercules, CA). On average, a petri dish (3 cm diameter; ~50.000 cells) yielded ~100 μg of total proteins and after subfractionation ~2–3 μg of synaptosomal proteins. Before electrophoresis, synaptosomes were resuspended in lysis buffer. Various lysis buffers were tested: a) 8 M urea, 4% CHAPS, 100 μl resuspension volume; b) 2% SDS, 100 l resuspension volume; c) 1% SDS in 100 mM PBS, 100 μl resuspension volume; d) 10% Zwittergent 3–10 (Calbiochem, Milano), 100 μl resuspension volume; e) 10% Zwittergent 3–10, 1 ml resuspension volume. In most experiments the lysis buffer (e) (10% Zwittergent 3–10, 1 ml resuspension volume) was used because it yielded the best protein display. Synaptosomal and cytosolic proteins were then precipitated by cold acetone (80% final concentration; 2 hr, -20°C) followed by centrifugation at 8000 g for 30 min (4°C). Protein pellets were then washed twice with acetone 80%, dried under a flow of nitrogen and then resuspended in 350 μl of 6 M urea, 2 M thiourea, 4% CHAPS, 2% IPG-buffer 3–10 NL (GE Healthcare, Milan, Italy), 65 mM DTT, 0.05% Bromophenol blue. At this stage sample protein concentration was re-determined.

### Gel electrophoresis

Proteins were separated by 2D gel electrophoresis. Protein samples were applied to 18 cm IPGstrips pH 3–10 NL (GE Healthcare) by in-gel rehydration (1 hr at 0 V followed by 8 hrs at 30 V; 20°C). Analytical 2D gels were loaded with 100 μg of proteins while preparative gels, for mass spectrometry identification of protein spots, were loaded with 1 mg of proteins (equivalent to 50–500 culture Petri dishes/gel). Focusing was performed with an IPGphore system (GE Healthcare) at 50 μA max per IPG strip with a gradient voltage (8000 V max) for a total of 65 kVh. Strips were equilibrated for 18 min in 0.5 mM pH 6.8 Tris-HCl buffer containing 6 M urea, 30% glycerol, 2% SDS, 0.05% Bromophenol blue and 2% DTT, then for 5 min in the same buffer containin 2.5% iodacetamide in place of DTT. The strips were then transferred onto 9–16% gradient acrylamide SDS-PAGE gels (20 × 20 cm, × 1.5 mm) for the second dimension separation (20 mA per gel at 14°C). Protein spots in analytical and preparative gels were visualized by highly sensitive silver staining [30] or MS compatible silver stain [31] respectively. Apparent Mr was estimated by comparison with values obtained from molecular weight reference markers (Precision, Bio-Rad). *pI *values were calibrated as described in the GE Healthcare guide lines for estimation of *pI *values for 18 cm IPG pH 3–10NL. Most of the reagents here used were from Sigma (St. Louis, MO). Zwittergent 3–10 was obtained from Calbiochem.

### Western Blotting

Proteins from total homogenate and synaptosomial fraction resolved on 10% acrylamide SDS-PAGE and some of the preparative 2DE gels were electrotransferred to nitrocellulose sheets (tris-glycine methanol buffer, 70 mA, overnight at 4°C) and immunolabelled with some specific antibodies against well known synaptic markers (the same nitrocellulose sheets were probed several times with different synaptic antibodies). The antibodies used in these experiments were: anti glutamate receptors GluR1 (rabbit policlonal made in house, 1:500); anti-P38 (Synaptic System, 1:500): anti-synaptotagmin-1 (rabbit policlonal against the lumenal domain, made in house, 1:1000); anti-VAMP-2, anti-synaptophysin, anti-syntaxin1A, anti-rab3A and anti-Chromogranin A (Synaptic system,1:500); anti-sec8 (Stressgene), anti-βActin and anti-βtubulin (Sigma, 1:1000). Antibodies reactivity was revealed by using appropriate HRP-conjugated secondary antibodies followed by chemiluminescence reaction and film exposure. By Ponceau red staining of nitrocellulose membranes and comparison with protein spots visualized in the silver-stained gels, antibodies reactivity was used as landmarks to precisely put in frame the reference 2D map.

### Gel Data Analysis

Stained gels were scanned at high resolution using a Personal SI Laser densitometer (Molecular Dynamics, Sunnyvale, CA). The resulting 2D patterns were analyzed using Progenesis PG240 v2006 software (Nonlinear dynamics, Newcastle, UK). Statistical analysis was performed using GraphPad Prism 4, V4.03 software (GraphPad Inc., SanDiego, CA). The statistical analysis of protein expression changes (evaluated as normalized spot volume) was performed by using unpaired Student's t-test with two-tailed p value. In all analysis, p < 0.05 was considered to be statistically significant. To apply a more stringent and reliable statistical criteria to the analysis of expression changes, we considered only changes affecting those spots that could be reliably identified in all gels belonging to the same group. This criterion was applied only after automatic spot detection which was run together in all gels according to a combined warping and matching tool available in the PG240 software. Furthermore, only statistically significant differences showing a variation above 1.5-folds were considered as relevant. To assess the signal to noise ratio of the single experiment, quality related information was extracted from gel images. In particular, the distribution of the grey intensity levels of the pixels belonging to the background, as excluded from the areas segmented as protein spots, was calculated as well the intensity distribution for the spot peaks. The juxtaposition of the two density functions, estimated from the occurrence frequencies of the intensity levels, provides a qualitative measurement of how the signal emerges from the image background. For a comparison of the spatial distribution of the detected spots in gel images corresponding to different cellular compartments, a two colors map was adopted, where positions are considered in terms of *pI *and MW, with a resolution of 0.1 *pI *and 1 kDa. The histograms of the relative abundance of detected spots along each dimension, for both the compared channels were obtained from the linearized map.

### Protein identification

Protein spots were visualized by staining preparative gels with MS compatible silver stain [32]. Spots of interest were excised from gels, reduced, alkylated and digested overnight with bovine trypsin (Roche), as previously described [31]. After tryptic digestion of the fractionated/resolved proteins, the resulting peptides mixtures were analyzed using MALDI-TOF MS.

### MALDI-TOF MS analysis

One μL aliquots of the supernatant were used for MS analysis using the dried droplet technique and CHCA as matrix. MS spectra were obtained on a MALDI-TOF Voyager-DE STR (Applied Biosystem) mass spectrometer. Alternatively, when no clear identification was obtained, acidic and basic peptide extraction from gel pieces after tryptic digestion was performed (with ammonium bicarbonate 50 mM and 50% acetonitrile or 10% Formic acid and 50% acetonitrile). The resulting peptide mixtures subjected to a single desalting/concentration step before MS analysis over Zip- TipC18 (Millipore Corporation, Bedford, MA, USA). Spectra were internally calibrated using trypsin autolysis products and processed via Data Explorer software. Proteins were identified by searching a comprehensive non-redundant protein database using both ProFound and Mascot programs [33,34] for cross validation of protein identifications. One missed cleavage per peptide was allowed, and an initial mass tolerance of 50 ppm was used.

## List of Abbreviations

Two-dimensional poliacrylamide gel electrophoresis, 2DE; Matrix-assisted laser desorption ionization time of flight, MALDI-TOF; Mass spectrometry, MS; Post-Translational Modifications, PTM.

## Authors' contributions

VC carried out 2D-electrophoresis. YS-R performed mass spectrometry analysis. GP set up lysis conditions. AB prepared neuronal cultures and purified synaptosomes. CVC, LP and SC, performed image analysis. AB participated in mass spectrometry analysis and manuscript preparation. MA participated in 2DE analysis and manuscript preparation. AM conceived the study and drafted the manuscript. All authors read and approved the final manuscript.

## Supplementary Material

Additional file 1 **Synaptic protein enrichment assayed by Western blots analysis**. Synaptic protein enrichment assayed by Western blots analysis of obtained loading total homogenate (Tot) and purified synaptosomes (Syn) from CA3-CA1 hippocampal cultures (50 μg/lane), same method and source material used for synaptic 2D gels. Proteins were resolved on a 10% acrylamide SDS-PAGE and electrotransferred to nitrocellulose for immunolabelling with specific antibodies against well known synaptic markers Antibodies used: Anti glutamate receptors GluR1 (rabbit policlonal made in house, 1:500), P38 (Synaptic System, 1:500) synaptotagmin-1 (rabbit policlonal against the lumenal domain, made in house, 1:1000), VAMP-2 (Synaptic system,1:500), Beta-tubulin (Sigma, 1:1000). Antibodies reactivity was revealed by using appropriate HRP-conjugated secondary antibodies followed by chemiluminescence reaction and film exposure. Click here for file

Additional file 2 List of differentially expressed proteins in the synaptosomal *vs*. crude cytosolic fractions. Click here for file

Additional file 3 Statistical analysis of proteins present with different expression level in synaptosomal and crude cytosolic fractions. Click here for file
