# Supplementary figures and images for: Protein fingerprints of cultured CA3-CA1 hippocampal neurons: comparative analysis of the distribution of synaptosomal and cytosolic proteins
Source: BMC Neurosci. 2008 Apr 10;9:36. doi: 10.1186/1471-2202-9-36 (PMC2324106; doi:10.1186/1471-2202-9-36)

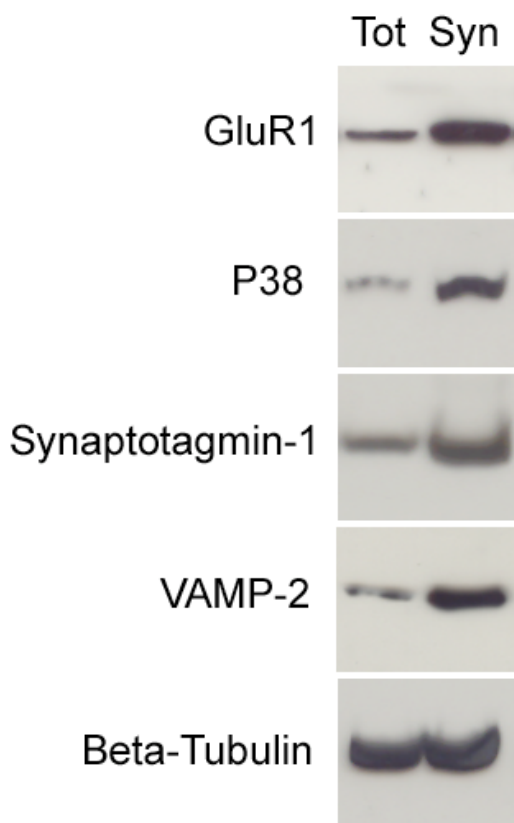

Supplement: Additional file 1 — Synaptic protein enrichment assayed by Western blots analysis. Synaptic protein enrichment assayed by Western blots analysis of obtained loading total homogenate (Tot) and purified synaptosomes (Syn) from CA3-CA1 hippocampal cultures (50 μg/lane), same method and source material used for synaptic 2D gels. Proteins were resolved on a 10% acrylamide SDS-PAGE and electrotransferred to nitrocellulose for immunolabelling with specific antibodies against well known synaptic markers Antibodies used: Anti glutamate receptors GluR1 (rabbit policlonal made in house, 1:500), P38 (Synaptic System, 1:500) synaptotagmin-1 (rabbit policlonal against the lumenal domain, made in house, 1:1000), VAMP-2 (Synaptic system,1:500), Beta-tubulin (Sigma, 1:1000). Antibodies reactivity was revealed by using appropriate HRP-conjugated secondary antibodies followed by chemiluminescence reaction and film exposure. [file 1471-2202-9-36-S1.pdf]
