# Supplementary material for: Protein fingerprints of cultured CA3-CA1 hippocampal neurons: comparative analysis of the distribution of synaptosomal and cytosolic proteins
Source: BMC Neurosci. 2008 Apr 10;9:36. doi: 10.1186/1471-2202-9-36 (PMC2324106; doi:10.1186/1471-2202-9-36)
Supplement: Additional file 2 — List of differentially expressed proteins in the synaptosomal vs. crude cytosolic fractions. [file 1471-2202-9-36-S2.doc]

| Additional file 2 | | | |
| --- | --- | --- | --- |
|  | | | |
| **List of differentially expressed proteins in the synaptosomal *vs.* crude cytosolic fractions** | | | |
| spot # | Accession number* | Protein name | Functional group |
| **Detected only in cytosol** | | |  |
| 119 | P13668 | Stathmin | CK |
| 70 | P25809 | Creatine kinase, ubiquitous mitochondrial [Precursor] | M |
| 90 | P04764 | Alpha-enolase | M |
| 128 | P62775 | Myotrophin | Sy |
| 97 | P46462 | Transitional endoplasmic reticulum ATPase | T |
| 102 | P07901 | Heat shock protein [Fragment] | PF |
| 108, 109 | P04905 | Glutathione S-transferase Yb-1 | PF |
| 107 | P04904 | Glutathione S-transferase Yc-1 | PF |
| 112 | P08010 | Glutathione S-transferase Yb-2 | PF |
| 111 | P08009 | Glutathione S-transferase Yb-3 | PF |
| 116 | P14942 | Glutathione S-transferase alpha-4 | PF |
| 94 | Q63347 | 26S protease regulatory subunit 7 | PF |
| 91 | Q9JHW0 | Proteasome subunit beta type 7 [Precursor] | PF |
| 118 | P01041 | Cystatin B | O |
| 12 | O35264 | Platelet-activating factor acetylhydrolase IB subunit beta | O |
| **Detected only in synaptosomes** | | |  |
| 48 | Q61274 | Alpha-cardiac actin [Fragment] | CK |
| 22 | P37805 | Neuronal protein 25 | CK |
| 34, 35 | P04636 | Malate dehydrogenase, mitochondrial [Precursor] | M |
| 8, 85 | Q99NA5 | NAD+specific isocitrate deihydrogenase a-subunit | M |
| 29 | P07825 | Synaptophysin | Sy |
| 30 | P32851 | Syntaxin 1A | Sy |
| 46 | P37377 | α-synuclein | Sy |
| 45 | Q63754 | β-synuclein | Sy |
| 73 | P25286 | Vacuolar proton translocating ATPase 116 kDa subunit a isoform 1 | Sy |
| 66 | P50516 | Vacuolar ATPsynthase catalytic subA | I |
| 28 | Q62824 | Exocyst complex component Sec8 | T |
| 17 | Q00981 | Ubiquitin carboxyl-terminal hydrolase isozyme L1 | PF |
| 52 | Q9DB15 | 60S ribosomal protein L12,mitochondrial | PF |
| 24 | P10354 | Cromogranina A E | S |
| 10 | P60766 | Cell division control protein 42 homolog | S |
| 62 | P62994 | Growth factor receptor bound protein 2 | S |
| 11 | P31399 | ATP synthase D chain, mitochondrial | M |
|  | | | |
| **Higher expression levels in synaptosomes** | | |  |
| 36 | P04691 | Tubulin beta chain | CK |
| 49 | P11240 | Cytochrome c oxidase polypeptide VA | M |
| 16 | P19234 | NADH-ubiquinone oxidoreductase 24 kDa subunit | M |
| 9 | P31044 | Phosphatidylethanolamine-binding protein | Sy |
| 6 | P52555 | Endoplasmic reticulum protein ERp29 | PF |
| 99 | P10111 | Peptidyl-prolyl cis-trans isomerase A | PF |
| 43 | P11598 | Protein disulfide isomerase A3 | PF |
| 121 | P63102 | 14-3-3 protein zeta/delta | S |
| 13 | P67779 | Prohibitin | O |
| 95 | Q8CDL9 | RIKEN full-length enriched library, clone:4931419P11 | Uk |
| 96 | P10719 | ATP synthase beta chain, mitochondrial | M |
| **Higher expression levels in crude cytosol** | | |  |
| 18 | P04797 | Glyceraldehyde-3-phosphate dehydrogenase | M |
| 105 | P04797 | Glyceraldehyde-3-phosphate dehydrogenase | M |
| 27 | P04764 | Alpha enolase | M |
| 83 | P42123 | L-lactate dehydrogenase B chain | M |
| 69 | P09606 | Glutamine synthetase | M |
| 122 | P07335 | Creatine kinase, B chain | M |
| 114 | P48500 | Triosephosphate isomerase | M |
| 38 | P63039 | 60 kda heat shock protein, mitochondrial precursor (hsp60) | PF |
| 115 | P17220 | Proteasome subunit alpha type 2 | PF |
|  |  |  |  |
| CK, cytoskeletal and their interacting proteins; M, metabolism; SY, synaptic components; I, receptors, ion channel and adhesion molecules; T, vescicle transport and recycling; PF, protein fate (synthesis, folding, modification and destination); S, signalling proteins; O, other; Uk, unknown; * Swiss-Prot database. | | | |
