# Supplementary material for: Protein fingerprints of cultured CA3-CA1 hippocampal neurons: comparative analysis of the distribution of synaptosomal and cytosolic proteins
Source: BMC Neurosci. 2008 Apr 10;9:36. doi: 10.1186/1471-2202-9-36 (PMC2324106; doi:10.1186/1471-2202-9-36)
Supplement: Additional file 3 — Statistical analysis of proteins present with different expression level in synaptosomal and crude cytosolic fractions. [file 1471-2202-9-36-S3.doc]

| Additional file 3 | | | | | | | |
| --- | --- | --- | --- | --- | --- | --- | --- |
|  | | | | | | | |
| List and statistical analysis of differentially expressed proteins in synaptosomal (S) *vs.* crude cytosolic (C) fractions | | | | | | | |
|  | | | | | | | |
|  |  | **S** | | **C** | |  |  |
| **Spot**  **#** | **Protein name** | **m**  **(Vol Norm)** | **SD** | **m**  **(Vol Norm)** | **SD** | **P**  **(student’ t)** | **Fold of**  **increase** |
|  | | | | | | | |
| **Higher expression levels in Synaptosomes** | | | | | | | |
| 36 | Tubulin beta chain | 5,48 | 3,2 | 0,07 | 0,08 | 0,043 | 75,1 |
| 49 | Cytochrome c oxidase polypeptide VA | 5,2 | 2,65 | 0,69 | 0,32 | 0,042 | 7,6 |
| 16 | NADH-ubiquinone oxidoreductase 24 kDa subunit | 4,10 | 1,92 | 0,91 | 0,51 | 0,049 | 4,5 |
| 9 | Phosphatidylethanolamine-binding protein | 3,73 | 0,93 | 1,7 | 0,82 | 0,046 | 3,1 |
| 6 | Endoplasmic reticulum protein ERp29 | 0,67 | 0,34 | 0,07 | 0,07 | 0,041 | 8,6 |
| 99 | Peptidyl-prolyl cis-trans isomerase A | 2,46 | 1,34 | 0,27 | 0,18 | 0,049 | 8,9 |
| 43 | Protein disulfide isomerase A3 | 0,77 | 0,4 | 0,11 | 0,08 | 0,049 | 6,6 |
| 121 | 14-3-3 protein zeta/delta | 1,14 | 0,47 | 0,36 | 0,1 | 0,047 | 3,1 |
| 13 | Prohibitin | 2,40 | 1,37 | 0,13 | 0,09 | 0,045 | 17,5 |
| 95 | RIKEN full-length enriched library, clone:4931419P11 | 0,34 | 0,13 | 0,11 | 0,04 | 0,042 | 3 |
| 96 | ATP synthase beta chain, mitochondrial | 4,74 | 1,49 | 1,52 | 1,3 | 0,047 | 3,1 |
|  |  |  |  |  |  |  |  |
| **Higher expression levels in crude cytosol** | |  |  |  |  |  |  |
| 18 | Glyceraldehyde-3-phosphate dehydrogenase | 0,23 | 0,23 | 0,88 | 0,27 | 0,034 | 3,8 |
| 105 | Glyceraldehyde-3-phosphate dehydrogenase | 2,28 | 1,62 | 5,59 | 1,19 | 0,046 | 2,5 |
| 27 | Alpha enolase | 2,00 | 0,96 | 4,07 | 0,58 | 0,033 | 2 |
| 83 | L-lactate dehydrogenase B chain | 0,55 | 0,34 | 2,06 | 0,87 | 0,048 | 3,7 |
| 69 | Glutamine synthetase | 0,46 | 0,39 | 1,41 | 0,37 | 0,037 | 3,1 |
| 122 | Creatine kinase, B chain | 2,00 | 1,16 | 4,40 | 0,47 | 0,029 | 2,2 |
| 114 | Triosephosphate isomerase | 0,38 | 0,08 | 0,82 | 0,26 | 0,049 | 2,2 |
| 38 | 60 kda heat shock protein, mitochondrial precursor | 3,50 | 1,11 | 0,48 | 0,37 | 0,011 | 8,6 |
| 115 | Proteasome subunit alpha type 2 | 0,22 | 0,11 | 0,73 | 0,29 | 0,047 | 3,3 |
|  |  |  |  |  |  |  |  |
| m: mean of optical density indicated as normalized spot volume; Vol Norm: normalized spot volume; SD: standard deviation. | | | | | | | |
